# Supplementary material for: Double Discourse: Qualitative Perspectives on Breast Screening Participation among Obese Women and Their Health Care Providers
Source: Int J Environ Res Public Health. 2019 Feb 13;16(4):534. doi: 10.3390/ijerph16040534 (PMC6407106; doi:10.3390/ijerph16040534)
Supplement: Supplementary file 1 [file ijerph-16-00534-s001.pdf]

Figure 1. Female obese participants interview guide: Understanding breast screening participation among obese women in Western Sydney.

Consent

Reiterate:

Confidentiality procedures,
Research process,
Withdrawing from research,
Stopping recording of the interview,
Encourage questions.

Demographic questions:

Age:
Occupation:
Postcode:
Marital status:
Children:
Level of education:
Country of birth:
Cancer history:
BMI:

Breast screening questions:

I just want to start off with you telling me about your experiences with any breast screening that you have had and how you feel about that screening. In other words it is your chance to tell me what you think/know about breast screening and anything that may have encouraged you or stopped you from having the recommended screening...

Prompts:

How important do you think breast screening is?
What breast screening have you had in the past?

If have had screening:

Why did you decide to have that screening?
What issues did you have to overcome to have the screening? *Prompt physical, emotional?*
Who did you talk to about the screening – prompt – family, friends, clinician – why did you talk to these people?
How did these people influence you to have the screening?
What healthcare experiences have you had in the past that may have influenced your screening decision?
What other regular screening do you have?

At the mammogram appointment:

What information were you given about how much the mammogram before it started?
How did you feel about the information you were given?
What other more information would you have liked to receive?
What opportunities did you have to ask questions?

How did comfortable you feel when you were having the mammogram?
How did you feel when the mammogram was finished?
Was there anything that you expected to happen that did not? *Prompt did you receive any unasked for advice about losing weight?*
What could the staff have done to make you feel more comfortable/uncomfortable?

If haven't had screening:

Why did you decide not to have that screening? *Prompt was there a specific fear e.g. pain/embarrassment?*
What issues were there to you having the screening? *Prompt physical, emotional?*
Who did you talk to about the screening – prompt – family, friends, clinician – why did you talk to these people?
How did these people influence your decision to not have the screening?
What healthcare experiences have you had in the past that may have influenced your screening decision? *Prompt insensitive comments made by healthcare providers*
What other regular screening do you have?

Diabetes/Obesity questions:

How long have you have you been overweight for?
How often do you need to visit the doctor for this condition?
How does your doctor make you feel about your weight?
What advice does your doctor give you about your condition?
How does your condition affect your healthcare experiences in general?
How does you doctor encourage you to have breast screening?

Risk questions:

Are you aware of any increased health risks due to being overweight
(Prompts: cancer as such as endometrial cancer, heart disease, diabetes complications)?

Final questions:

How do you feel about continuing on with breast screening in the future?
Do you feel you have the information and support for your future screening?
What could be done to make getting mammograms more pleasant?
What do you think could be done to improve healthcare for women like yourself Prompt social media?
Is there anything you would like to ask me?

Figure 2. Provider Interview Guide: Understanding breast screening participation among obese women Western Sydney.

Consent

Reiterate:

Confidentiality procedures,
Research process,
Withdrawing from research,
Stopping recording of the interview,
Encourage questions.

Demographic questions:

Age:
Role:
Time in role:
Postcode:
Country of birth:

In this interview we would like to focus on the experiences you have had in the past of screening women who are obese. Could you please think about specific patients and patient encounters and describe them to me?

Prompts:

Can you tell me about one patient that you had a good encounter with/one that was a challenge?
What have you done in the past to make someone who is obese feel comfortable?
How do you feel about having to screen obese women?

Can you tell me about a very obese patient you performed a mammogram on, what difficulties did you have doing the exam itself? *Prompt equipment*
What equipment do you have that is designed to accommodate obese patients? *Prompt armless waiting room chairs, extra large gowns, large wheelchairs*

Have you come across women who are not up to date with their screening?
What do you think has prevented this person from having regular screening?
What reasons do those patients offer for not getting regular screening?
What do you say to these women about their screening? *Prompt how do you encourage them to screen?*
